#  Morbimortalidade materna no Brasil e a urgência de um sistema nacional de vigilância do *near miss* materno

**DOI:** 10.1590/0102-311XPT013923

**Published:** 2023-08-07

**Authors:** Michelle Elaine Siqueira Ferreira, Raquel Zanatta Coutinho, Bernardo Lanza Queiroz

**Affiliations:** 1 Universidade Federal de Minas Gerais, Belo Horizonte, Brasil.

**Keywords:** Near Miss, Saúde Materna, Sistema de Vigilância em Saúde, Morte Materna, Healthcare Near Miss, Maternal Health, Health Surveillance System, Maternal Death, Near Miss Salud, Salud Materna, Sistema de Vigilancia Sanitaria, Muerte Materna

## Abstract

A Organização Mundial da Saúde (OMS) recomenda a análise dos casos de morbidade materna severa/*near miss* materno como complemento às análises das mortes de mães, dado que a incidência é mais elevada e os fatores preditivos dos dois desfechos são semelhantes. Tendo em vista que as razões de mortalidade materna, no Brasil, têm se mantido constantes apesar do compromisso firmado durante a Assembleia Geral da Organização das Nações Unidas (ONU), em 2015, o objetivo deste artigo é propor um sistema nacional de vigilância de *near miss* materno. Propõe-se a inclusão dos eventos *near miss* materno na Lista Nacional de Notificação Compulsória de Doenças, Agravos e Eventos de Saúde Pública, por meio da compatibilização dos critérios diagnósticos de *near miss* materno, informados pela OMS, com os códigos da Classificação Internacional de Doenças (CID) para identificação dos casos. Tendo em vista que a vigilância em saúde se faz baseada em diversas fontes de informações, a notificação poderia ser feita pelos profissionais dos serviços de saúde tão logo fosse identificado um caso confirmado ou suspeito. A partir do estudo dos fatores associados aos desfechos, espera-se a avaliação mais qualificada dos serviços voltados à assistência obstétrica e consequente implementação de políticas mais eficientes de prevenção não apenas do óbito materno, mas de eventos que podem tanto causar sequelas irreversíveis à saúde da mulher quanto aumento do risco de óbito fetal e neonatal.

## Abstract (en)

The World Health Organization (WHO) recommends the analysis of severe maternal morbidity/maternal near miss cases as complementary to the analysis of maternal deaths since the incidence is higher and the predictive factors of the two outcomes are similar. Considering that the reasons for maternal mortality in Brazil have remained constant despite the commitment made during the General Assembly of the United Nations in 2015, this article aims to propose a nationwide maternal near miss surveillance system. We propose the inclusion of maternal near miss events in the National List of Compulsory Notification of Diseases, Injuries, and Public Health Events, via the compatibility of the diagnostic criteria of maternal near miss, informed by the WHO, with the codes of the International Classification of Diseases for the identification of cases. Considering that health surveillance is based on several sources of information, notification could be made by health service professionals as soon as a confirmed or suspected case is identified. With the study of the factors associated with the outcomes, we expect a qualified evaluation of the services focused on obstetric care and consequent implementation of more efficient policies to prevent not only maternal death but also events that can both cause irreversible sequelae to women’s health and increase the risk of fetal and neonatal death.

## Abstract (es)

La Organización Mundial de la Salud (OMS) recomienda el análisis de los casos de morbilidad materna grave/*near miss* materno como complemento a los análisis de las muertes maternas, dado que la incidencia es más elevada y los factores predictivos de los dos resultados son similares. Teniendo en vista que las razones de mortalidad materna, en Brasil, se han mantenido constantes a pesar del compromiso firmado durante la Asamblea General de la Organización de las Naciones Unidas, en el año 2015, el objetivo de este artículo es proponer un sistema de vigilancia de *near miss* materno de alcance nacional. Se propone la inclusión de los eventos de *near miss* materno en la Lista Nacional de Notificación Obligatoria de Enfermedades, Agravios y Eventos de Salud Pública, por medio de la compatibilización de los criterios diagnósticos de *near miss* materno; informados por la OMS, con los códigos de la Clasificación Internacional de Enfermedades para identificación de los casos. Teniendo en vista que la vigilancia en salud se basa en diversas fuentes de Informaciones, la notificación podría ser hecha por los profesionales de los servicios de salud, tan pronto fuese identificado un caso confirmado o sospechoso. Se espera que el estudio de los factores asociados a los resultados conduzca a una evaluación más calificada de los servicios de atención obstétrica y a la consecuente implementación de políticas más eficientes de prevención no solo de la muerte materna; sino de eventos que pueden tanto causar secuelas irreversibles a la salud de la mujer como aumento del riesgo de muerte fetal y neonatal.

## Introdução

Desde meados da década de 1990, as razões de mortalidade materna (RMM) globais declinaram cerca de 44%, porém com grandes diferenças entre países ^1^^,^^2^. Nos países em desenvolvimento, apesar de terem registrado diminuições no referido indicador, alguns ainda apresentam altas razões de mortes maternas relacionadas, em boa parte, às causas evitáveis ^2^. O Brasil experimentou um importante declínio em seu indicador, passando de 72,4 óbitos maternos por 100 mil nascidos vivos em 2009 para 57,9 óbitos maternos por 100 mil nascidos vivos em 2019. No entanto, observa-se um importante diferencial entre as regiões brasileiras. No último ano citado, enquanto o Pará, na Região Norte, e o Piauí, na Região Nordeste, registraram RMM de 96,1 e 98,1 por 100 mil nascidos vivos, Santa Catarina, na Região Sul, e o Distrito Federal, na Região Centro-oeste, registraram RMM de 30,6 e 21,2 por 100 mil nascidos vivos, respectivamente ^3^.

Desde a Conferência do Cairo (Egito), ocorrida em 1994, quando ficou pactuado que os países deveriam se esforçar para reduzir suas RMM em 75%, são empregados grandes esforços para garantir a melhoria da assistência prestada à gestante, puérperas e seus bebês e, em consequência, reduzir os óbitos maternos. Ao longo das últimas três décadas e das demais conferências de população, esses esforços globais levaram à adoção de medidas mais eficientes para a promoção da saúde materna. A literatura aponta que a redução dos óbitos maternos é resultado de melhorias nas políticas públicas com medidas para diminuição das iniquidades em saúde, especialmente ampliação do acesso ao pré-natal, qualificação da assistência ao parto e cuidados pós-parto ^1^^,^^4^.

Embora o Brasil tenha, na maior parte do período citado, se empenhado para garantir melhorias na assistência obstétrica e neonatal, como a adoção de um sistema nacional de vigilância de óbitos maternos e a implementação de um programa nacional voltado ao pré-natal e ao nascimento, a já extinta Rede Cegonha ^5^, os ganhos no declínio das razões de mortalidade materna têm se mantido estagnados nos últimos anos ^3^ e muito acima do estabelecido para o país conforme os Objetivos de Desenvolvimento Sustentável (ODS): o patamar de 30 óbitos para cada 100 mil nascidos vivos até 2030 ^3^. Desses óbitos, cerca de 67% decorreram de causas obstétricas diretas, que são as complicações obstétricas durante a gravidez, o parto ou o puerpério devido a intervenções, omissões, tratamento incorreto ou eventos resultantes dessas causas ^3^^,^^6^.

No Brasil, a *Portaria nº 1.119* de 2008 ^7^ do Ministério da Saúde tornou obrigatória a investigação dos óbitos maternos e de mulheres em idade fértil. Em síntese, a obrigatoriedade tem como objetivos tanto a redução da subnotificação das mortes maternas quanto a análise dos fatores associados ao desfecho, como possíveis falhas na assistência obstétrica. Ainda que o Sistema de Vigilância de Óbitos Maternos tenha possibilitado avanços na redução da RMM brasileira e o estudo da dinâmica de saúde materna no país, os indicadores apontam, além dos diferenciais regionais, diferenciais sociodemográficos ^3^^,^^8^. Por outro lado, tendo em vista que, em números absolutos, a morte materna é considerada um evento raro, o que não desconsidera sua importância, e em alguns contextos não permite uma análise robusta dos fatores associados ao óbito, a OMS recomenda a incorporação da morbidade materna grave e do *near miss* materno nas investigações relacionadas à saúde materna ^1^^,^^9^.

O *near miss* materno ocorre quando uma mulher, devido a complicações provocadas ou agravadas por seu estado gravídico-puerperal, quase morre, não evoluindo para a morte em razão de intervenções eficazes da equipe de assistência à saúde ^9^^,^^10^. Nesse contexto, as vantagens de um sistema de vigilância de *near miss* materno estão associadas à possibilidade da análise da eficácia e efetividade das intervenções adotadas que impediram o óbito materno, à oportunidade de obter informações sobre a assistência e outros fatores associados ao evento junto à mulher que o experimentou, assim como pelo fato de a incidência de *near miss* materno ser mais alta que a de óbito oportunizar informações mais específicas, principalmente em regiões menos populosas.

O objetivo deste artigo é propor um sistema de vigilância nacional de *near miss* materno a ser implementado com base na inclusão desses eventos na Lista de Agravos de Notificação Compulsória e, visto que a vigilância em saúde é feita por fontes de informações diversas, discutir as possíveis bases utilizadas pela literatura. Neste trabalho, primeiramente apresentamos os critérios utilizados para a padronização dos conceitos de morbidade materna grave e *near miss* materno, seguido da apresentação das vantagens dos registros e avaliação desses eventos. Em seguida, apresentamos alguns exemplos de sistemas de vigilância de morbidade materna grave e *near miss* materno registrados no mundo e no Brasil. Por fim, sugerimos a implementação de um sistema brasileiro de vigilância de *near miss* materno. 

##  Morbidade materna grave e *near miss* materno: definições 

A saúde materna abrange as condições de saúde da mulher durante a gestação, o parto e o período puerperal. De acordo com Say et al. ^9^, embora a maioria das mulheres experimente uma gestação sem quaisquer intercorrências que possam comprometer sua saúde ou a de seus bebês, outras poderão evoluir para complicações em um continuum de eventos adversos provocados ou agravados por sua condição gravídico-puerperal. As complicações, nesse contexto, podem ser classificadas como baixo risco de morte, condições potencialmente ameaçadoras à vida, incluindo a morbidade materna grave e, por fim, os desfechos maternos graves, como *near miss* e o óbito materno ^9^. Em todos os casos, tendo em vista que mesmo uma mulher saudável pode evoluir para óbito, os cuidados assistenciais à gestante e à puérpera são essenciais para que qualquer intercorrência seja identificada em tempo hábil e tratada adequadamente para que a evolução para um desfecho materno grave seja evitada.

Conforme a OMS ^10^, considerado o compromisso assumido por diversas nações para melhoria da saúde materna e infantil, não apenas os óbitos devem ser reduzidos, mas as demais condições que podem causar sequelas irreversíveis à saúde sexual e reprodutiva das mulheres. Assim, em 2011, com a participação dos membros da Rede de Vigilância de Morbidade Materna Grave ^11^^,^^12^^,^^13^ e de outros profissionais e pesquisadores de diversos países, a OMS publicou um guia com recomendações para avaliação da qualidade do cuidado nas complicações maternas graves com abordagem voltada para o *near miss* materno ^10^^,^^14^. Para tanto, buscando padronizar os conceitos e, dessa forma, operacionalizar a adequada identificação dos casos, o documento traz algumas definições importantes, como a de complicações maternas graves, de intervenções críticas e de *near miss* materno, que explicaremos nos próximos parágrafos. 

As complicações maternas graves são definidas como condições potencialmente ameaçadoras à vida e, ainda que o guia adote apenas cinco como critérios de inclusão inicial - hemorragia pós-parto grave, pré-eclâmpsia grave, eclâmpsia, sepse/infecção sistêmica grave, rotura uterina -, elas abrangem outras condições clínicas, tais como gravidez ectópica, descolamento prematuro de placenta, edema pulmonar e parada respiratória ^9^. Já as intervenções críticas são aquelas imprescindíveis para o manejo das condições maternas que, caso não fossem adotadas, a mulher evoluiria para óbito, sendo as principais: transfusões sanguíneas, radiologia intervencionista e intervenções cirúrgicas de emergência na cavidade abdominal, como histerectomias, excluindo as cesáreas ^9^^,^^10^.

O *near miss* materno, por seu turno, após ampla revisão da literatura especializada em saúde materna pelo grupo de trabalho da OMS sobre mortalidade materna e classificações de morbidade, é definido como uma mulher que quase morreu, mas sobreviveu a uma complicação que ocorreu durante a gravidez, parto ou dentro de 42 dias após o término da gravidez ^9^^,^^10^^,^^14^. Embora o conceito pareça amplo e de difícil identificação, os casos de *near miss* são aqueles associados às disfunções orgânicas apresentadas pela mulher e que foram provocadas ou agravadas pela gestação, ou por intercorrências durante e após o parto. Nesse contexto, com base nos critérios diagnósticos existentes na literatura ^15^^,^^16^^,^^17^^,^^18^ associados às diversas definições anteriores à formalização pela OMS ^9^^,^^19^, o grupo de trabalho apresentou uma lista de condições ameaçadoras à vida, todas associadas a alguma categoria de disfunção orgânica dos sistemas circulatório, respiratório, nervoso central, renal, hepático, entre outros.

Para identificar as disfunções orgânicas capazes de levar uma parturiente ou puérpera a óbito, foram elencados 25 critérios, divididos em três grupos: clínico, laboratorial e de manejo. Os critérios clínicos são compostos por 11 condições, entre elas, a falha de coagulação, o choque e a perda de consciência superior ou igual a 12 horas. Os laboratoriais, compostos por oito critérios, incluem a presença de glicose e cetoácidos na urina, a saturação de oxigênio abaixo de 90% por 60 minutos ou mais e a trombocitopenia aguda. Por fim, os critérios baseados em manejo incluem, entre os seis estabelecidos, a diálise para insuficiência renal aguda, a histerectomia após infecção ou hemorragia e a ressuscitação cardiopulmonar ^9^. Nesse contexto, caso a mulher preencha um ou mais critérios entre os 25 e não evolua para óbito, ela será considerada um caso de *near miss* materno ^9^.

Uma vez definidos os conceitos, é importante entender as vantagens dos registros e avaliação desses eventos, como destacado por alguns estudos que investigaram a incidência de *near miss* e outras complicações maternas graves. Com relação à escala, isto é, obter um número maior de eventos em comparação aos óbitos maternos, a estratégia é útil para ter maior robustez na análise, descobrir problemas de assistência rapidamente e inserir controles sociodemográficos, tendo em vista que a maioria dos óbitos está relacionada à falência de órgãos ^20^^,^^21^^,^^22^. Há, também, a possibilidade de ouvir a sobrevivente e obter a sua perspectiva sobre os fatores de riscos e a inadequação do cuidado, além de quantificar os requerimentos para uma unidade de terapia intensiva e calcular taxas de incidência dos eventos de maior letalidade. 

Por exemplo, em um estudo que quantificou a incidência de quatro das principais causas de morbimortalidade materna na Irlanda - hemorragia, distúrbios hipertensivos, sepse e trombose -, Leitao et al. ^23^, baseados no modelo iceberg ^15^, entre 2009 e 2019, identificaram 1.029 casos de *near miss* materno para cada óbito materno associado à hemorragia; 58 casos de *near miss* por cada óbito materno associado à eclâmpsia, 92 casos de *near miss* materno por cada óbito associado à sepse e 35 casos de *near miss* por cada óbito materno associado à trombose. 

Embora os óbitos maternos, por ser o desfecho mais grave associado à saúde da mãe, recebam maior atenção, a vigilância de outros eventos, capazes de causar sequelas irreversíveis à saúde sexual e reprodutiva da mulher, é incipiente ou, em muitos países, negligenciada ^9^^,^^23^^,^^24^. Dada a incidência dos eventos, as consequências para as famílias e para a sociedade, além das implicações financeiras para o sistema de saúde, é fundamental que os fatores associados aos desfechos obstétricos sejam investigados. Uma vez que os casos de *near miss* são similares aos de mortalidade materna, eles podem ser utilizados para fornecer informações sobre o cuidado obstétrico, assim como para identificar obstáculos e melhores práticas diante de complicações agudas, permitindo que ações corretivas sejam colocadas em prática de forma pontual e que a prioridade seja dada aos casos mais urgentes ^1^^,^^25^. Assim, o guia e as demais recomendações da OMS buscam não apenas indicar ferramentas e meios para reduzir a morbimortalidade materna, mas direcionar os serviços de saúde para que, por meio de evidências, realizem avaliações periódicas dos procedimentos e técnicas para que as mulheres não apenas sobrevivam, mas experimentem um ciclo gravídico-puerperal com risco reduzido de desfechos maternos graves, ou seja, *near miss* e óbitos maternos ^10^. Na próxima seção, são apresentados alguns exemplos de sistema de vigilância da morbidade materna grave e *near miss* materno.

##  Sistemas de vigilância de morbidade materna grave e *near miss* materno no mundo e no Brasil 

Tendo em vista as dificuldades que alguns países enfrentam para alcançar as metas assumidas durante as Conferências das Nações Unidas ocorridas nos últimos 30 anos, que estabeleceram que as mortes maternas globais não poderão ultrapassar 70 mortes por 100 mil nascidos vivos até 2030, a Organização das Nações Unidas (ONU) definiu estratégias para alcance dos compromissos assumidos pelos países signatários. No que concerne à saúde materna, destaca-se a necessidade de investimentos em intervenções baseadas em evidências robustas capazes de proporcionar uma análise eficiente e confiável dos determinantes dos processos saúde-doença. Nesse contexto, ainda que o óbito seja evento sentinela para avaliação e monitoramento da qualidade da assistência obstétrica, a OMS e a comunidade científica recomendam a implementação de sistemas de vigilância em morbidade materna grave e *near miss* materno ^1^^,^^23^^,^^26^, em complemento aos sistemas de vigilância do óbito, dado que a morte representa apenas uma parcela de desfechos maternos graves. Sobre esse aspecto, a literatura guarda alguns exemplos de tentativas de monitoramento e avaliação de casos de complicações maternas graves nos quais os dados foram obtidos em auditorias hospitalares, sistemas de informação e inquéritos populacionais, exemplificados a seguir.

Por meio da análise de prontuários de cuidado na unidade de terapia intensiva (UTI) obstétrica e pelo registro de atendimento hospitalar, em um estudo para o Suriname, Verschueren et al. ^27^ analisaram a associação dos casos de *near miss* com a ocorrência de morte perinatal. Outras tentativas de se estabelecer investigações de *near miss* com base em coletas de prontuário e formulários específicos em hospital, como parte do protocolo de atendimento ou em auditorias, são relatadas nas revisões de Okusanya et al. ^28^, Geller et al. ^1^^,^^16^, Knight et al. ^21^, van Dillen et al. ^29^ e Bashour et al. ^30^. Na sua maioria, utilizavam-se os formulários padronizados para identificar mulheres que sofreram as complicações segundo os critérios definidos, podendo ser feitos de forma retrospectiva (apenas as que sofreram) ou prospectiva, em que todas são registradas e as que sofreram respondem questionário adicional.

Além de estudos pontuais, alguns países contam com Sistemas Nacionais de Vigilância Epidemiológica de eventos graves associados à gravidez, ao parto e ao puerpério. Na China, o Sistema Nacional de Vigilância de *Near Miss* Materno (NMNMSS) foi criado em 2010 e utiliza os indicadores da OMS para o monitoramento materno e perinatal, com leves modificações ^31^^,^^32^. O NMNMSS cobre cerca de 400 unidades em 30 províncias chinesas e, com base em formulários padronizados, são coletados dados sociodemográficos, informações sobre complicações durante a gestação, abortamentos, intervenções e desfechos perinatais e maternos. Após análise do protocolo médico, as informações são passadas para as fichas individuais da mulher e digitadas em uma plataforma *online* localizada no Escritório Nacional de Vigilância da Saúde Materno-Infantil da China (NOMCHS). Diante desse desenho, que coleta informações no momento da entrada no hospital, é possível calcular até mesmo a mortalidade específica por causa também associada ao *near miss*, incluindo diferentes complicações e disfunções orgânicas ^31^.

O Reino Unido, que conta com um sistema de vigilância de óbito materno desde a década de 1950, instituiu o Sistema de Vigilância Obstétrica do Reino Unido (UKOSS), cobrindo todas as unidades obstétricas do país e permitindo a análise, seja em auditorias ou em outros tipos de estudos, de uma gama de eventos associados à gravidez, neles incluídos o *near miss* materno ^21^. Destaca-se que, buscando eficiência do sistema, as condições investigadas são dinâmicas e a abordagem é feita por tópicos para considerar os problemas emergentes e de maior impacto em cada período. De acordo com Knight et al. ^21^, essa categoria de abordagem permite que novos estudos sejam introduzidos quando há questões clínicas específicas a serem abordadas, além de evitar a fadiga da coleta de dados e minimizar a carga de coleta de informações para os médicos que relatam. Nessa perspectiva, foi possível a introdução, em tempo hábil, de estudos sobre as gestantes internadas com H1N1, em 2009, assim como a investigação dos casos de COVID-19 em gestantes, desde março de 2020, quando a pandemia foi decretada pela OMS ^33^.

No caso do Brasil, Carvalho et al. ^34^, com base nos dados disponibilizados no Sistema de Informações Hospitalares do SUS (SIH-SUS), identificaram cerca de 1,06 milhão de casos de *near miss* materno, entre 2000 e 2012, sendo as razões mais altas observadas nas regiões Norte e Nordeste. Um estudo, que também utilizou os dados do SIH-SUS, identificou 766.249 casos de *near miss* materno entre as 20.891.040 internações por causas obstétricas, registradas entre 2010 e 2018 ^35^. Como pode ser visto, assim como em outros países, a morte materna, ainda que elevada, representa uma pequena parcela de todas as complicações vivenciadas por parturientes e puérperas brasileiras. Assim, considerando a alta incidência de *near miss* materno, a vigilância contínua dos casos pode contribuir não apenas para a construção e melhoria das políticas voltadas à redução da morte materna, mas também para entendimento e identificação dos fatores associados ao número de mulheres que continuam experimentando morbidade materna mesmo após o avanço da cobertura da assistência profissional ao pré-natal, ao parto e ao pós-parto.

No Brasil, a criação do Sistema de Vigilância do Óbito Materno ocorreu recentemente, em 2009, o que possibilitou acompanhar a evolução desse indicador com mais segurança somente a partir dessa data. No entanto, Leal et al. ^36^, com base nas estimativas do projeto Carga Global de Doenças, do Instituto de Métricas e Avaliação em Saúde, Estados Unidos (IHME), apontam uma tendência de declínio na RMM brasileira, ainda que caracterizada por uma grande variabilidade regional nos níveis e nos ritmos de variação entre 1990 e 2019. Segundo os resultados do estudo, no período, apenas 14 das 27 unidades federativas apresentaram reduções consistentes nas RMM, das quais somente cinco observaram reduções acima de 50%: Pernambuco (-74,6%), Bahia (-66%), Rio Grande do Norte (-56,7%), Minas Gerais (-55,8%) e Alagoas (55%). Em contrapartida, no Tocantins a redução foi de apenas 0,1% e no Amapá houve aumento de cerca de 9% ^36^. 

Em momentos de crise sanitária e consequente sobrecarga dos serviços de saúde, esses indicadores podem sofrer importantes efeitos. Durante a pandemia de COVID-19, estima-se que a RMM tenha alcançado cerca de 71,97 óbitos por 100 mil nascidos vivos em 2020 e 107 mortes maternas por 100 mil nascidos vivos em 2021 ^37^. Um estudo que comparou o excesso de mortalidade materna nas regiões brasileiras, entre março de 2020 e maio de 2021, encontrou valores exorbitantes em todas as regiões (63%-89%). No entanto, o valor observado permaneceu sempre mais alto do que o esperado na Região Norte, em todos os trimestres avaliados, seguido da Região Nordeste, alto em dois trimestres avaliados ^38^. Portanto, considerados os diferenciais socioeconômicos entre as regiões e os grupos populacionais, reforça-se a necessidade de vigilância contínua para avaliação e adequação das necessidades em saúde materna segundo o contexto sanitário, político e socioeconômico, uma vez que as RMM estimadas nos dois primeiros anos de pandemia sugerem que esse grupo se torna ainda mais vulnerável em contextos de crise. 

A literatura tem avançado nas análises de diversas fontes de informações para mapear os fatores associados aos indicadores de saúde materna e se reconhece a importância da vigilância do óbito materno, que já conta com um sistema obrigatório de investigação em todo o território nacional. Uma vez que os processos de investigação em saúde não são excludentes, mas complementares, dadas as experiências disponíveis na literatura ^11^^,^^39^^,^^40^^,^^41^^,^^42^^,^^43^^,^^44^, observa-se que o Brasil tem tanto potencial para implementação de um sistema nacional de vigilância de *near miss* materno quanto emergência em sua implementação. Na próxima seção discutimos uma proposta para sua efetivação.

##  Proposta de um sistema brasileiro de vigilância de *near miss* materno 

A vigilância em saúde é uma importante ferramenta para planejamento e avaliação das ações voltadas à saúde da população, uma vez que fornece informações coletadas em processos sistemáticos e contínuos acerca dos fatores determinantes e condicionantes de saúde individual ou coletiva ^45^^,^^46^. Recomenda-se que diversas fontes de dados sejam utilizadas para obtenção das informações relevantes para fins de identificação e acompanhamento de eventos relacionados à saúde ^46^^,^^47^.

No caso brasileiro, a base do sistema de vigilância é formada pela notificação compulsória de doenças e agravos à saúde, em que as informações são registradas no Sistema de Informação de Agravos de Notificação (SINAN). Além do SINAN, os principais sistemas de informações utilizados para vigilância em saúde são o Sistema de Informação sobre Mortalidade (SIM), Sistema de Informação sobre Nascidos Vivos (SINASC) e o SIH-SUS. Outras fontes de dados são registros hospitalares, inquéritos domiciliares, investigações de surto, estudos epidemiológicos e estudos conduzidos por seguimentos da sociedade civil, desde que permitam a identificação imediata do problema e seu enfrentamento de forma eficiente e em tempo oportuno ^47^^,^^48^.

No contexto da saúde materna, ainda que a vigilância de óbitos maternos desempenhe importante papel na investigação dos fatores associados às mortes de mulheres no ciclo gravídico-puerperal, na correção dos dados e no fornecimento de subsídios para avaliação e melhoria da assistência obstétrica, os resultados podem não ser representativos de toda a população, pois pode haver subnotificação ou incompletude. Por outro lado, por mais que seja urgente a adoção de medidas eficientes que impeçam a morte de uma mulher, é necessário que eventos maternos graves também sejam prevenidos dadas as possíveis sequelas físicas e psicológicas infligidas à mulher. Em síntese, a investigação de um óbito materno possibilita intervenções preventivas de novos óbitos, mas para a mulher que morre nada pode ser feito, mesmo que muito possa ser apreendido. No caso do *near miss* materno, a possibilidade é que se aprenda tanto com as falhas da assistência quanto com os acertos capazes de impedir o desfecho grave, com a diferença de que as informações complementares que não são possíveis de captação por meio de dados institucionais podem ser obtidas junto à mulher.

Alguns estudos realizados no Brasil indicam direcionamentos possíveis para a implementação da vigilância em morbidade materna. Entre julho de 2009 e junho de 2010, um estudo transversal multicêntrico foi realizado em 27 hospitais, das cinco regiões do país, pela Rede Nacional de Vigilância de Morbidade Materna Grave. Com base nas definições recomendadas pela OMS ^9^, o grupo objetivou validar os critérios diagnósticos do *near miss* materno considerando sua característica preditiva do óbito materno. Tendo como pressuposto que uma mulher que experimenta um evento de *near miss* é exatamente como aquela que morre, exceto pelo desfecho, as análises confirmaram os critérios estabelecidos com uma sensibilidade de 100% e especificidade de 92% ^11^^,^^44^^,^^48^. Nesse sentido, além de ter contribuído para a elaboração do guia para manejo dos casos de complicações maternas graves publicado pela OMS em 2011 ^10^, o estudo indica que é possível a investigação dos casos de *near miss* materno por meio de auditorias hospitalares.

Outro caminho indicado pela literatura é a utilização de dados secundários extraídos do SIH-SUS. Ainda que sua função não seja de vigilância, mas contábil-financeira, o sistema oferece informações sobre causas de internação e procedimentos realizados que podem ser utilizadas para identificar casos de *near miss* e outras complicações maternas graves ^34^^,^^35^^,^^49^^,^^50^^,^^51^. Dois estudos utilizaram essa metodologia para analisar a incidência do *near miss* materno para todas as macrorregiões do Brasil. Nakamura-Pereira et al. ^49^ adotaram a compatibilização dos critérios diagnósticos estabelecidos pela OMS com os códigos da Classificação Internacional de Doenças, 10ª revisão (CID-10) para analisar a capacidade do SIH-SUS na captação dos casos. Herdt et al. ^35^, tendo em vista que a compatibilização dos critérios diagnósticos propostos por Mantel et al. ^15^ e Waterstone et al. ^17^ captam a maior parte das condições potencialmente ameaçadoras à vida, compatibilizaram os códigos da CID-10 com esses critérios para medir a incidência do agravo entre 2010 e 2018. De acordo com os achados, foi observada uma tendência de aumento das razões no período analisado, sendo as regiões Norte e Nordeste aquelas com os maiores indicadores.

Conforme explicitado, é possível investigar as ocorrências de *near miss* materno tanto pelo uso de dados primários como de dados secundários. Contudo, a literatura pontua algumas limitações. No caso de auditorias hospitalares, as investigações demandam o empenho das equipes de saúde no registro adequado das informações, sendo necessário, entretanto, assumir o pressuposto de que os protocolos de manejo e de assistência são seguidos adequadamente, uma vez que há critérios de intervenção específicos para identificação dos casos. Além disso, as auditorias são mais dispendiosas quanto ao investimento financeiro e à disponibilidade de tempo ^11^. 

No que se refere ao SIH-SUS, por ser uma fonte secundária, o registro inadequado das informações pode gerar resultados não condizentes com a realidade. Estudo realizado em um hospital do Estado do Rio de Janeiro, por exemplo, mostrou baixa eficiência na captação dos casos de *near miss* materno ^49^. No entanto, o SIH-SUS tem passado por constantes avaliações e melhorias ao longo dos anos, sendo as variáveis utilizadas para identificação das causas de internação classificadas como sem dados faltantes e com baixa discrepância nos dados ^52^. Por esse motivo, havendo a possibilidade de buscar os casos por causa de internação e de procedimentos realizados, de acordo com a avaliação do Instituto de Pesquisa Econômica Aplicada (IPEA), ainda que o SIH-SUS não seja um sistema de vigilância em saúde, como o SIM e o SINASC, ele pode ser uma importante fonte de informação para a vigilância de *near miss* materno, assim como já é utilizado para a vigilância de outras morbidades.

A *Portaria nº 1.271* de 2014 ^45^, do Ministério da Saúde, define doença como enfermidade ou estado clínico, independente de origem ou fonte, que represente ou possa representar um dano significativo para os seres humanos (Art. 2º, III). Considerando que o *near miss* materno se enquadra no conceito de doença para os fins a que se destina a referida norma, a sugestão deste presente artigo é que o evento seja incluído na Lista de Notificação Compulsória para que, diante disso, seja obrigatório, via SINAN, o comunicado de casos suspeitos ou confirmados. Quanto à responsabilidade de toda a equipe profissional, destaca-se que o procedimento de notificação pode ser executado pelos três níveis de atenção à saúde: primário, secundário e terciário. 

Um possível entrave para a inclusão do *near miss* materno no rol de doenças e agravos de importância nacional é a ausência de código específico na CID. No entanto, tendo em vista a existência de instrumento que já é utilizado em alguns estados do país, considerando os critérios padronizados pela OMS, os casos podem ser identificados a partir da análise do quadro clínico da mulher ainda no hospital, ou por meio das informações constantes no sumário de alta hospitalar, durante a consulta puerperal, por exemplo. 

Com base em sistemas implementados em outros países, destacando-se os de vigilância chinês e do Reino Unido ^32^^,^^33^, as instituições de saúde ou pessoas responsáveis pela atenção ao parto, inclusive os profissionais de saúde, poderiam utilizar um formulário padrão com quesitos a serem preenchidos pela parturiente ou puérpera com informações sobre permanência prolongada, necessidade de terapia intensiva, transfusão sanguínea, entre outros critérios indicativos de complicação materna grave, assim como as causas subjacentes a condições ameaçadoras à vida e desfechos maternos graves ^10^. No caso deste último, o exemplo emblemático é o aborto, que deve ser listado, juntamente como outros transtornos, doenças ou complicações do manejo. 

Os abortos, ainda que sejam responsáveis por grande parte da morbidade materna, ainda são subnotificados, possivelmente devido à criminalização de sua prática. Em muitos casos, declarados como infecção puerperal, hemorragias ou sepses, a sua notificação adequada possibilitaria tanto dimensionar a magnitude do número de abortos inseguros, relacionados a um maior risco de morbimortalidade materna, assim como permitiria a avaliação da qualidade da atenção às suas complicações, mesmo entre os abortos espontâneos. Cumpre esclarecer que, tendo como base o Formulário Individual de Coleta de Dados para *near miss* materno, padronizado pela OMS, o aborto foi incluído na ferramenta de investigação como causa subjacente aos desfechos maternos graves ^9^^,^^10^.

Conforme discutido em Ayres et al. ^46^ e assegurado pelas normas nacionais que abordam a Vigilância em Saúde (*Lei nº 8.080* de 1990; *Lei nº 6.259* de 1975; e *Constituição Federal* de 1988), as investigações não se exaurem em uma única fonte de informação. Nesse contexto, considerada a potencialidade do SIH-SUS para identificação de diversas morbidades ^52^, ele tem potencial como fonte complementar para análise do *near miss* materno. Assim, como a técnica de associação dos códigos da CID-10, adotada por Nakamura-Pereira et al. ^49^, incluindo suas atualizações, aos critérios diagnósticos recomendados pela OMS, exemplificados anteriormente, mecanismos de identificação dos casos podem ser elaborados tanto para investigação quanto para aplicação em modelos preditivos de novos casos. A partir desses mecanismos, é possível vincular a vigilância dos casos de *near miss* materno à vigilância dos óbitos maternos transformando o sistema existente em um sistema de vigilância de morbimortalidade materna ou, em outra possibilidade, ao incluí-lo na Lista de Agravos de Notificação Compulsória, possibilitar que todos os casos sejam reportados e, assim, sejam utilizados não apenas para otimizar a vigilância do óbito, mas possibilitar um amplo acesso aos interessados no assunto, tanto no âmbito científico quanto no de utilização para melhoria das políticas voltadas à saúde materna.

Com relação à viabilidade de sua implementação, como os custos operacionais, fluxos, prazos e instrumentos, e a capacidade institucional de levá-lo adiante, além das limitações já expostas e os desafios ainda enfrentados pelo Sistema de Vigilância de Óbito, a criação e execução de um sistema complementar almeja a análise de uma equipe multidisciplinar do governo, da sociedade civil e de especialistas. No entanto, o Brasil tem investido na melhoria dos sistemas de investigação e da qualidade das notificações, o que pode reduzir os custos com treinamento, manutenção e investimento em novas tecnologias. 

## Considerações finais

O *near miss* materno, conforme discutido, ocorre quando uma mulher apresenta disfunções orgânicas provocadas ou agravadas pela gravidez, por eventos adversos durante o parto ou no puerpério, mas não evolui para óbito apesar da gravidade de seu quadro de saúde. Embora seja associado à qualidade da assistência hospitalar, uma vez que o óbito materno foi evitado, está associado a piores desfechos neonatais e sequelas irreversíveis à saúde sexual e reprodutiva de mulheres que o experimentam.

Duas perspectivas podem ser discutidas no que diz respeito à incidência do *near miss* e à necessidade de implementação de um sistema de vigilância contínuo em adição ao Sistema de Vigilância de Óbito Materno. A primeira diz respeito ao fato de que é inegável que a assistência obstétrica adequada previne a evolução de gestações com risco habitual ou com complicações com baixo risco de morte para desfechos maternos graves ^9^. Tendo em vista que as causas evitáveis da morbimortalidade materna são mais prevalentes e, considerando que para cada óbito há uma infinidade de mulheres que experimentam o *near miss*, esses casos podem indicar os fatores associados tanto à evolução do quadro de saúde da mulher para um *near miss* materno quanto à evitabilidade do óbito. Nesse contexto, a investigação pode fornecer ferramentas para criar protocolos de intervenção eficientes para replicação em casos semelhantes, assim como podem contribuir para a adequação de protocolos obsoletos ou não baseados em evidências científicas. 

A segunda perspectiva que se apreende dos casos de *near miss* materno diz respeito aos direitos sexuais e reprodutivos de toda mulher, que deve abranger esforços máximos de uma experiência gestacional e puerperal positiva. Ainda que a morte materna, assim como o óbito fetal e neonatal, sejam eventos maternos trágicos, a experimentação de um *near miss* materno pode acarretar não apenas sequelas físicas, mas emocionais e psicológicas importantes. É notório que as medidas adotadas para redução do óbito proporcionam, em consequência, melhoria da qualidade assistencial e redução dos riscos maternos de modo geral. No entanto, mesmo em países em que as razões de morte materna são baixas, o *near miss* materno ainda é uma preocupação, uma vez que a saúde das mães deve estar sob constante vigilância, dada a complexidade do espectro de morbidade materna que pode evoluir de uma gestação sem complicações para um desfecho grave, caso a gestante não seja monitorada de forma adequada por toda a gestação, no parto e no puerpério. 

O Brasil, embora tenha avançado na melhoria de seus indicadores maternos, não conseguiu cumprir a meta assumida, durante a Cúpula do Milênio, para redução da RMM em 75% até 2015 e tem mantido sua RMM, nos últimos anos, em cerca de 60 óbitos maternos por 100 mil nascidos vivos, cerca de duas vezes o valor esperado para 2030, de acordo com compromisso assumido pelo ODS ^3^. Assim, um sistema de vigilância de *near miss* materno pode contribuir para aperfeiçoamento das políticas e serviços de saúde nas regiões onde a RMM é mais baixa, proporcionando cada vez mais a redução de outros desfechos, como a morbidade materna grave e severa e a redução da morbimortalidade fetal e neonatal. Afinal, em países citados neste artigo ^32^^,^^33^, a RMM em 2017 se aproximou de 17 na China e 7,4 no Reino Unido ^53^. Ademais, as evidências obtidas nessas localidades podem contribuir para aperfeiçoamento das investigações nas regiões onde os indicadores ainda são preocupantes tanto na aplicação das técnicas de vigilância quanto no aprimoramento dos protocolos assistenciais. Embora as desigualdades em saúde sejam uma realidade brasileira e a complexidade de fatores envolvidos em cada região requer intervenções adaptadas ao contexto territorial, acredita-se que o primeiro passo para traçar uma estratégia política, nesse sentido, esteja relacionado à garantia da robustez dos dados. Assim, enfatizando a importância do Sistema de Vigilância de Óbito Materno, a vigilância do *near miss* materno pode contribuir para a elucidação de falhas e acertos na assistência obstétrica, possibilitando, dessa forma, avaliação e adequação, cada vez mais minuciosa, da política e dos serviços voltados à saúde materna.
